# Food allergy in the Saudi population: trends, triggers, and strategies for care – a systematic review

**DOI:** 10.3389/fpubh.2026.1849220

**Published:** 2026-06-30

**Authors:** Mohammed S. Alshammari

**Affiliations:** Department of Medical Laboratories, College of Applied Medical Sciences, Shaqra University, Shaqra, Saudi Arabia

**Keywords:** allergen labeling, epinephrine auto-injector, food hypersensitivity, milk allergy, Saudi Arabia, sesame allergy

## Abstract

Food allergy is a common clinical problem in Saudi Arabia with multiple factors contributing to it including the changes in lifestyle, especially among the younger generation. This systematic review presents evidence regarding the epidemiology, etiology, and management of allergic diseases in the Kingdom. This systematic review with narrative synthesis was conducted following PRISMA 2020 guidelines. Due to substantial heterogeneity in study design, diagnostic approaches, and outcome measures, findings were synthesized narratively according to evidence type and research focus., The review used 20 studies and regulatory documents obtained through a systematic search of PubMed/MEDLINE, Scopus, and the Saudi Digital Library. The food allergy in Saudi Arabia are similar to those in other countries with common allergens such as milk and eggs, but regional foods such as sesame and seafood are also responsible for allergic reactions. Elimination diets in children with food allergy may increase the risk of nutritional deficiencies. In Saudi Arabia, where vitamin D deficiency is already prevalent, careful nutritional monitoring is recommended. Despite the allergen labeling and awareness campaigns by the Saudi Food and Drug Authority (SFDA), food allergies remain a major problem in clinical practice. Several reports are available on the prevalence and awareness of food allergies, but the findings are dependent on self-reported data.

## 1 Introduction

The allergic reactions to food are increasing throughout the world due multiple factors that includes dietary patterns, environmental exposure, microbiome changes, and genetic susceptibility (1). The allergic reactions can be minor, such as a skin rash, or life-threatening anaphylaxis (2). The allergic reactions in the Saudi population are different from the patterns reported in Western countries. The environmental factors and ancestry in Saudi population influences food allergy (3). Apart from this, vitamin D deficiency also contributes to food allergy (4). Also, reduced microbial exposure and alterations in the microbiome, especially in Saudi cities, may influence immune responses and allergy risk (5).

Food allergies in Saudi Arabia are a mixture of allergens identified worldwide, along with local factors. In young children, the main allergy triggers are the same as in many other countries, such as cow’s milk, eggs, and peanuts. However, the local diet introduces important differences. For example, sesame allergy is more common than in Western countries. This may be related to early and frequent dietary exposure to sesame-containing local foods like tahini and halwa. Regional food habits within the kingdom also contribute to food allergies. In coastal cities such as Jeddah and the Eastern Province, allergies to seafood (fish and shellfish) make up a large part of the allergy problem for adults (1), which is due to higher consumption. On the other hand, studies propose camel milk as a possible alternative to cow milk in children (6). The camel milk’s protein structure is different from that of cow’s milk, as it lacks a common allergen called beta-lactoglobulin. While some studies in Saudi Arabia have utilized IgG-based testing, it remains clinically discouraged for diagnosing food allergies as it reflects dietary exposure rather than true pathology.

This systematic review outlines the food allergies in Saudi Arabia, analyzing different factors that affect the prevalence rates. The difference between universal food allergies and the influence of genetic and dietary patterns found in Saudi Arabia is discussed. Finally, the patient care related to food allergy, the challenges faced in clinical management, and public awareness have been analyzed.

## 2 Methodology

### 2.1 Study design

This review followed the PRISMA 2020 systematic review guidelines (7). Various study designs available in the literature (cross-sectional surveys, retrospective case–control studies, prevalence studies, and commentary reviews) were used. This systematic review outlines the evidence on the epidemiology, etiology, clinical management, and public awareness of food allergy in the Saudi population. The review protocol was developed *a priori*; however, the review was not prospectively registered in PROSPERO.

#### 2.1.1 Review question (PICOS framework)

The review question was formulated using the PICOS framework:

- Population (P): Children and adults residing in Saudi Arabia.
- Intervention/Exposure (I): Exposure to food allergens, dietary factors, environmental influences, and food allergy management strategies.
- Comparator (C): Where available, comparisons between allergic and non-allergic individuals, different age groups, or different exposure patterns.
- Outcomes (O): Food allergy prevalence, allergen triggers, risk factors, clinical manifestations, nutritional impact, awareness levels, and management practices.
- Study design (S): Observational studies, prevalence studies, retrospective clinical studies, awareness surveys, review articles, and relevant regulatory documents.
- The review sought to answer the following question: “What is the current evidence regarding the epidemiology, risk factors, clinical characteristics, management, and awareness of food allergy in the Saudi population?”

### 2.2 Information sources and search strategy

A systematic literature search was conducted across three electronic databases: PubMed/MEDLINE, Scopus, and Web of Science. Google Scholar was used for supplementary grey-literature screening. The search was last updated on February, 2026. The reference lists of full-text articles were also screened for additional studies to include articles that could not be obtained through the database search (backward citation tracking). The following search terms were used in PubMed; (“Food Hypersensitivity”[MeSH] OR “food allergy” OR “food hypersensitivity”) AND (“Saudi Arabia”[MeSH] OR “Saudi Arabia” OR “Middle East”) AND (prevalence OR epidemiology OR incidence) with filters; Article language-English and Species-Humans. The Scopus search was done using TITLE-ABS-KEY(“food allergy” OR “food hypersensitivity”) AND TITLE-ABS-KEY(“Saudi Arabia” OR “Middle East”) AND TITLE-ABS-KEY (prevalence OR epidemiology OR incidence) with filters applied: document type-Article, Review and Language as English. The web of science topic search was carried out using the keywords as TS = (“food allergy” OR “food hypersensitivity”) AND TS = (“Saudi Arabia” OR “Middle East”) AND TS = (prevalence OR epidemiology OR incidence). The Google scholar search was performed using the keywrods ‘“food allergy” AND “Saudi Arabia” AND prevalence” and most relevant articles were screened. Only studies published in English were included. This restriction was applied because English is the predominant language of publication in the selected databases and because resources for translation of non-English studies were not available. While this approach may have introduced language bias, its impact is expected to be limited given the predominance of English-language biomedical publications in the region. Grey literature was screened through Google Scholar searches, backward citation tracking of included articles, and review of relevant Saudi Food and Drug Authority (SFDA) documents and international guideline publications. These sources were included to improve completeness of evidence capture and provide regulatory context.

### 2.3 Eligibility criteria

Studies were included if they met all of the following criteria: (1) reported original data or provided a review of food allergy in the Saudi population; (2) those reporting prevalence or incidence of food allergy, allergen triggers, clinical management strategies, or levels of knowledge and awareness among patients, caregivers, or healthcare workers; (3) published in English in peer-reviewed journals; and (4) conducted in Saudi Arabia or, in neighboring Gulf Cooperation Council countries (for comparison). Studies were excluded if they: (1) reported only non-food allergic conditions (e.g., respiratory allergy without dietary component); (2) were case reports in less than five participants; (3) were conference abstracts without full-text availability; or (4) focused solely on pre-clinical or *in vitro* methods without clinical outcomes. Regulatory documents from the Saudi Food and Drug Authority (SFDA) and international guideline bodies were also included.

### 2.4 Study selection process

**Figure 1:**
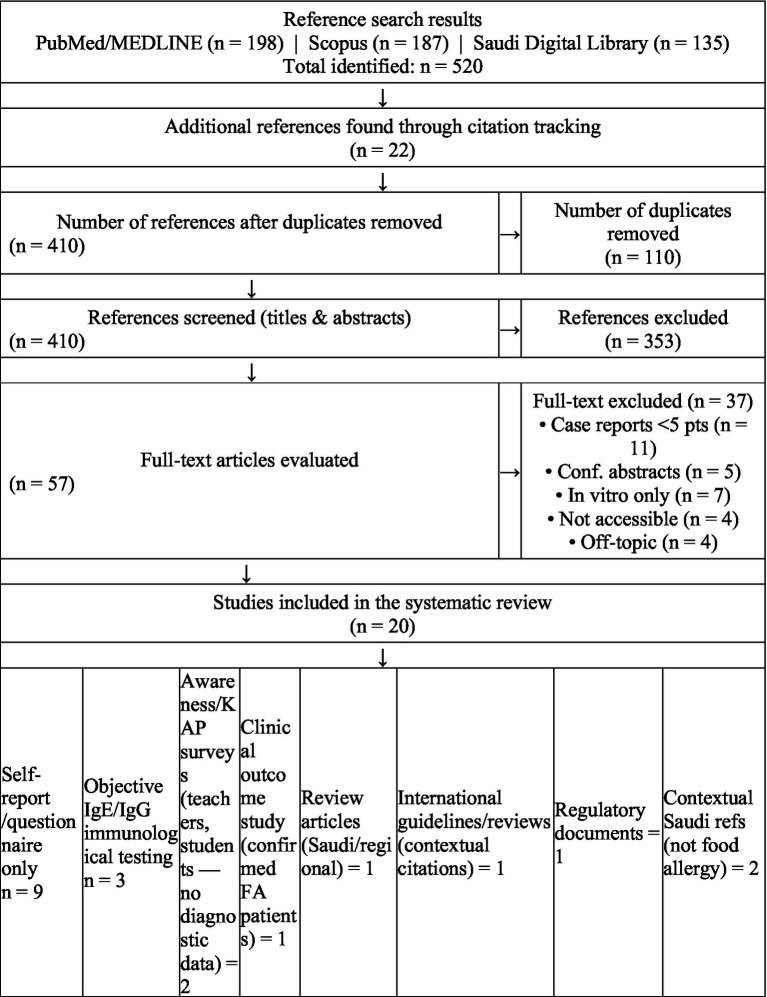
Food allergy in the Saudi population, search results.

Records retrieved from all databases were exported to a reference management tool and deduplicated. The selection process followed two sequential stages. In Stage 1, titles and abstracts of all identified records were screened against the predefined eligibility criteria; records not meeting any inclusion criterion were excluded. In Stage 2, full-text articles of all remaining records were obtained and assessed in detail. The full selection process, including records identified, screened, assessed for eligibility, and finally included, is summarized in Figure 1 (PRISMA flow diagram). In total, the database search yielded approximately 520 records. After deduplication (*n* = 110), 410 unique records were screened by title and abstract, of which 342 were excluded as irrelevant (non-Saudi population, non-food allergy, or duplicate findings). Sixty-eight full-text articles were assessed for eligibility, and 37 were excluded and a total of 20 peer-reviewed studies and regulatory documents, were included in this review (*n* = 20 sources). This is given in Table 1.

**Table 1:** Classification of included evidence sources and their contribution to the review.

Evidence type	Number of sources (*n*)	Primary contribution
Self-report/Questionnaire studies	9	Estimates of prevalence, reported allergens, risk factors, and patient experiences
Laboratory-based immunological studies (IgE/IgG)	3	Assessment of immunological reactivity and sensitization patterns
Awareness/KAP surveys	2	Evaluation of knowledge, attitudes, preparedness, and food allergy management practices
Clinical outcome studies	1	Clinical manifestations, nutritional impact, and patient outcomes
Review articles	1	Contextual synthesis of existing evidence and identification of knowledge gaps
International guidelines/Reviews	1	Diagnostic and management standards used for interpretation of findings
Regulatory documents	1	Food allergen labeling policies and public health regulations
Contextual Saudi references	2	Supporting evidence on related allergic conditions, environmental factors, and regional context
Total	20	—

KAP, Knowledge, attitude, and practice. Regulatory documents and review articles were included to provide contextual and policy-related evidence and were not used to estimate prevalence or clinical outcomes.

### 2.5 Data extraction and quality assessment

From each included study, the following data were extracted: first author and year of publication, study location within Saudi Arabia, study design, sample size, and population characteristics (age group, sex), primary outcome measures, key findings relevant to food allergy epidemiology or management, and reported limitations.

#### 2.5.1 Quality assessments of included studies

The methodological quality of included studies was assessed using a modified appraisal framework derived from the Joanna Briggs Institute (JBI) critical appraisal tools for observational studies (8). The assessment considered sampling methodology, representativeness of the study population, validity of outcome measurements, control of confounding factors, completeness of reporting, and appropriateness of statistical analyses.

Due to the heterogeneity of study designs included in this review, a simplified classification system was adopted (Table 1). Studies were categorized as:

- Low risk of bias: studies with clearly defined methodology, objective outcome measures, and adequate reporting.
- Moderate risk of bias: studies with generally acceptable methodology but limitations such as self-reported outcomes or incomplete control of confounding factors.
- High risk of bias: studies with substantial methodological limitations, unclear outcome definitions, or significant reporting deficiencies.

The quality assessment was used to guide the interpretation of findings during narrative synthesis rather than as an exclusion criterion.

### 2.6 Narrative synthesis of heterogenous evidence

The included studies exhibited substantial methodological and clinical heterogeneity with respect to study design, participant characteristics, diagnostic approaches, outcome measures, and evidence type. The evidence base comprised epidemiological surveys, retrospective clinical studies, laboratory-based immunological investigations, awareness and knowledge surveys, review articles, and regulatory documents. Owing to this heterogeneity, quantitative pooling of results or meta-analysis was not considered appropriate.

Therefore, a narrative synthesis approach was employed. Evidence was grouped according to research focus and evidence type, including: (1) epidemiology and prevalence studies, (2) pediatric and clinical studies, (3) laboratory-based immunological studies, (4) awareness and educational surveys, and (5) regulatory and policy initiatives. Findings from each category were synthesized separately before being integrated into the overall discussion. Summary tables (Tables 1, 2) were constructed to present the clustered evidence transparently, in line with PRISMA guidance for systematic review.

**Table 2:** Quality assessment of included studies.

Study type	Number of studies	Risk of bias
Self-reported prevalence surveys	9	Moderate–High
Awareness/KAP surveys	4	Moderate
Retrospective clinical studies	3	Moderate
IgE/IgG laboratory studies	3	Moderate
Clinical outcome studies with confirmed diagnosis	1	Low–Moderate
Regulatory/Guideline documents	Not applicable	Not assessed

IgE, immunoglobulin E; IgG, immunoglobulin G; KAP, knowledge, attitude, and practice; JBI, Joanna Briggs Institute.

## 3 Results

### 3.1 Overview of included evidence

The 20 included sources represented a diverse body of evidence. Nine studies were based primarily on self-reported questionnaires, three used laboratory-based immunological testing, four focused on awareness and educational outcomes, one investigated clinical outcomes in patients with confirmed food allergy, one was a review article, and two consisted of regulatory or contextual policy documents. Given these differences in methodology and objectives, findings are presented according to evidence type and interpreted within their respective methodological limitations.

### 3.2 Quality assessments of included studies

The quality assessment revealed significant variability in the methodological rigor of the included studies. Most were classified as having a moderate risk of bias, primarily due to their cross-sectional designs, reliance on self-reported data, and limited use of objective diagnostic methods. Questionnaire-based prevalence studies were particularly vulnerable to recall bias and outcome misclassification, as food allergy status was frequently based on participant perception rather than clinical confirmation.

In contrast, studies incorporating laboratory assessments such as skin prick testing or serum-specific IgE measurements offered a lower risk of bias regarding outcome ascertainment. However, several of these studies relied on food-specific IgG testing; because IgG testing is not recommended for diagnosing food allergies, these findings have limited clinical interpretability. Ultimately, only a small number of studies utilized clinically confirmed cases, which yielded a lower overall risk of bias.

Overall, the body of evidence was judged to be of moderate quality. Consequently, these findings should be interpreted with caution, particularly the prevalence estimates derived from self-reported surveys. To strengthen the evidence base for food allergy research in Saudi Arabia, future studies must prioritize standardized diagnostic criteria, objective allergy testing, and prospective designs (Table 2).

Risk of bias was assessed using a modified appraisal framework adapted from the Joanna Briggs Institute (JBI) critical appraisal tools for observational studies. Assessment domains included sampling methodology, population representativeness, validity of outcome measurement, management of confounding variables, completeness of reporting, and appropriateness of statistical analyses. Studies were categorized as Low Risk (meeting ≥80% of quality criteria), Moderate Risk (50–79%), or High Risk (<50%). The quality assessment was used to guide the interpretation of findings and was not employed as an exclusion criterion.

### 3.3 Epidemiology and prevalence of food allergy among adults in Saudi Arabia

Several studies have reported common food allergies and their management among adults in Saudi Arabia. A study from Jeddah found that roughly one in five adults reported a food allergy. The most common allergens reported in this group were eggs (22%), fruits (20.5%), and fish (13.8%). Interestingly, egg allergy in the region appears to follow a distinct local pattern. Unlike global trends, where it mostly affects infants, research from Jeddah identified it as common in adult males aged 20–40. These reactions were primarily triggered by egg whites and were often linked to other allergic conditions like rhinosinusitis and eczema. However, only very few participants knew that epinephrine is the emergency treatment for severe reactions (9). Another study from the same region involving over 1,600 individuals identified that foods like oats, barley, and cow’s milk showed higher IgG reactivity. While rates were similar between men and women, young children reacted to cow’s milk more frequently than adults (10). A study from the capital city of Riyadh on patients presenting with unexplained symptoms found high levels of IgG reactions to items like cola nut, brewer’s yeast, and wheat, with females showing a greater tendency for these sensitivities (11). However, it should be noted that IgG-based testing is not recommended for diagnosing food allergy and may reflect exposure rather than pathology.

Respiratory allergies, often connected to food allergies, also show high and varied prevalence. In Najran, asthma was diagnosed in over 27% of schoolchildren, with triggers including Bermuda grass and cat dander. Lifestyle factors like fast-food consumption and living near busy roads were also noted (12). In Qassim, nearly 29% of adults reported respiratory allergies, with family history being the strongest predictor. This high prevalence has a direct impact on daily life and safety (13). In Makkah, for instance, most food-allergic patients had experienced a life-threatening reaction, yet almost none carried epinephrine auto-injectors. Many expressed concern that public spaces like restaurants and schools are not prepared to assist during an allergic emergency (14). Similarly, at a university in the Eastern Province, nearly 10% of students had a diagnosed food allergy, but fewer than half consistently carried their prescribed epinephrine, often taking risks with allergen avoidance as they gained independence (15). A recent study by Khojah et al. on 2,100 parents across Saudi Arabia found that only 23.3% of affected children possessed life-saving epinephrine auto-injectors despite 26% of these children having asthma that significantly elevates the risk of fatal anaphylaxis (16). This low rate of emergency preparedness is particularly dangerous for the high-risk asthmatic group identified by Alsharairi, as comorbid asthma significantly elevates the risk of fatal anaphylaxis. Alsharairi (17) demonstrated that the transition toward a “Westernized” diet, which is high in processed fats and low in Vitamin D may be associated with “atopic march” in Saudi children, where food sensitivities progress eczema into chronic asthma. This nutritional shift increases the clinical impact of common local allergens like eggs, milk, and wheat, which are frequently linked to increased respiratory inflammation. Together, these studies highlight that while dietary interventions are essential for long-term prevention, urgent systemic changes are needed to ensure immediate access to life-saving medications for those already affected. A summary of these studies is presented in Table 3.

**Table 3:** Summary of key studies investigating food allergy in Saudi Arabia, grouped by research focus.

Research cluster	Included sources	Key data point (*n*)	Synthesis of findings	Clinical implications
Epidemiological baseline	Alharbi (2020) (9) and Alnahas (2023) (18)	8,942	Self-reported food allergy (20.2%) Respiratory allergies (up to 38.5%) Main reason reported was due to “Westernized” shifts.	National surveillance is hindered by high self-report bias.
High-risk behavioral gaps	Alosaimy (2022) (14), Hassan (2020) (15), and Khojah (2025) (16)	5,883	Despite high rates of anaphylaxis (68%), epinephrine carriage remains <5%.	Transition to independent life (university) increases lax avoidance behaviors.
Non-IgE hypersensitivity	Shakoor (2016) (11), Alkhateeb (2020) (10), and Atwah (2024) (19)	1,890	IgG reactivity to milk and staples (oats/wheat) in symptomatic patients.	Suggests a need to distinguish “exposure markers” from “pathology.”
Pediatric and atopic co-morbidities	Alqahtani (2016) (12) and Almatroudi (2021) (13)	2,550	34.5% of children had multiple allergic diseases Strong links to family history and “fast food.”	Urbanization and sterile environments (Hygiene Hypothesis) are primary drivers.

*n* (total sample size or number of participants); IgG, immunoglobulin G-associated with delayed food sensitivities. Westernized Shift is a dietary transition toward energy-dense, highly processed foods, which is identified as a primary driver of the region’s allergy epidemic.

### 3.4 Pediatric food allergy prevalence, growth impacts, and stakeholder awareness in Saudi Arabia

The growing issue of food allergies among children in Saudi Arabia shows important links to other health conditions and a need for better public understanding. A 2019 national survey of school-aged children found that reported allergy-related rhinitis was common and increased with age. Interestingly, diet appeared to play a role: frequently eating cooked vegetables was associated with roughly half the risk, while regular intake of foods like pulses and eggs was also protective for adolescents. Other factors, like monthly paracetamol use, cat ownership, and vigorous physical activity, were linked to higher allergy odds in teens (18).

The specific foods causing reactions show clear patterns. In a 2024 Jeddah clinic study, all children with allergic disorders showed elevated antibodies to at least one food. Dairy was the most common trigger, followed by gluten products and eggs. The study also found connections between specific allergies and conditions: atopic dermatitis was linked to dairy, gluten, and eggs; chronic hives to chicken and lamb; and asthma to certain fish. This suggests testing for these delayed food reactions could be useful in complex pediatric cases, despite ongoing debate about its role in standard care (19). Although food-specific IgG responses were observed, the clinical significance of these findings remains uncertain. Current allergy guidelines do not recommend IgG testing for diagnosing food allergy, and further studies are needed to clarify any potential clinical relevance.

Some observational studies suggest a higher prevalence of reported food sensitivities among children with autism spectrum disorder (ASD), although causal relationships remain unclear. A 2022 parent survey reported a prevalence of 23.4%, with cow’s milk and seafood as top triggers. Most reactions involved skin symptoms like redness and itching, occurring within minutes to hours after eating. A strong family history of allergies was seen among these families, pointing to shared immune factors that warrant further study (20).

For all allergic children, managing the condition carries its own risks. A 2023 Riyadh study of children with confirmed food allergies found that necessary dietary avoidance often led to impaired growth, particularly in boys. Removing staples like eggs, milk, and wheat negatively impacted height and weight, highlighting the urgent need for guided nutritional support to prevent stunting. These allergies are frequently connected to respiratory problems. Asthma is highly prevalent among Saudi children, and commentary has noted that food allergies and diets high in processed foods may worsen this inflammatory burden (4).

Effective management relies heavily on caregiver awareness, which currently has significant gaps. Studies of parents show moderate overall knowledge but persistent misconceptions and underuse of emergency medication like epinephrine. Awareness is a key factor that improves daily management, such as reading food labels and avoiding allergens. This knowledge gap extends to schools. A 2022 assessment of primary teachers found very positive attitudes toward helping allergic students but low knowledge of how to respond to a reaction. Few knew about emergency plans or epinephrine use, creating a dangerous delay in care during school hours (21). In another study, the primary school teachers in Al-Kharj, Saudi Arabia, displayed a highly positive attitude toward student safety but possessed dangerously low levels of practical knowledge and emergency skills. Most participants were unable to identify the clinical signs of anaphylaxis or the correct first-aid procedures required during a severe reaction. Furthermore, the survey revealed a widespread lack of familiarity with epinephrine auto-injectors, which are the primary medical intervention for life-threatening allergies (22).

The evaluation of level of food allergy awareness among parents in Saudi Arabia, identified significant gaps in emergency preparedness (23). The parents generally understood the seriousness of allergic conditions, but a substantial number lacked the practical skills needed to manage a life-threatening reaction. A primary concern identified was the widespread hesitation and lack of technical training regarding the administration of epinephrine auto-injectors. Additionally, many families were found to be unaware of the necessity for regular medication updates, often keeping expired devices that could fail during a crisis.

Further, studies show awareness among young adults is increasing. A 2023 study of university students, many future parents, found high awareness levels, especially when they had access to clear allergen information on menus. This supports public health measures like clear food labeling as an effective tool for prevention (24). The effectiveness of simulation-based training in improving food allergy management among elementary school staff in Rabigh region of Saudi Arabia reported that hands-on intervention drastically increased the readiness to use epinephrine auto-injectors from 18.8 to 84.5%. Participants showed significant improvements in identifying life-threatening symptoms, such as acute respiratory distress and facial swelling, which are often overlooked in theoretical training. The findings also highlighted a positive shift in teacher confidence, with most staff feeling better equipped to handle emergency anaphylaxis without immediate medical supervision (25).

The food allergies in Saudi children are prevalent and intertwined with other chronic conditions like asthma and ASD. While caregiver attitudes are positive, a lack of specific knowledge and preparedness at home and in schools poses a real risk. A summary of food allergy studies among pediatric population is summarized in Table 4.

**Table 4:** Comparative analysis of studies on food allergies in Saudi children and awareness among parents, teachers, and students.

Research focus	Primary sources (author, year)	Sample size (*n*)	Key findings and data trends	Awareness and management gaps
National respiratory and atopic trends	Alnahas et al. (2023) (18) and Alsharairi (2019) (17)	7,682+	Rhinitis prevalence 18.7–38.5% Asthma affects ~23% of children.	Regional variations suggest a need for national monitoring.
Nutritional and growth impact	Bin Obaid et al. (2023) (4)	72	Multi-allergen exclusion leads to lower height-for-age and BMI z-scores, primarily in boys (*p* ≤ 0.05).	Lack of nutritional counseling exacerbates stunting risks.
ASD and protein sensitivities	Alhuzimi and Alharbi (2023) (20)	125	23.4% FA prevalence in ASD children; proteins (milk/seafood) are dominant triggers.	Parental awareness mediates the link between knowledge and management.
Cow’s milk protein allergy (CMPA)	Alqurashi et al. (2025) (26) and Baghlaf et al. (2023, 2021) (27, 28)	475+	Sibling history is the strongest risk (OR = 10.07); 100% IgG reactivity to milk in some pediatric samples.	Only “intermediate” knowledge on practical milk alternatives. IgG food testing is not recommended; it indicates exposure, not allergy.
Educational and caregiver awareness	Alomran et al. (2022) (22), Alotaibi et al. (2020) (23), and Aljameel et al. (2023) (24)	1,611	Teachers show low practice scores (2.1/8); only 23.3% of parents carry an EAI.	Urgent need for mandatory school protocols and EAI stocking.

*n* (total sample size or number of participants); ASD, autism spectrum disorder; CMPA, cow’s milk protein allergy; EAI, epinephrine auto-injector; FA, food allergy; IgE, immunoglobulin E-associated with immediate allergic reactions; IgG, immunoglobulin G-associated with delayed food sensitivities; OR, odds ratio a measure of association where OR >1 indicates higher risk; SFDA, Saudi food and drug authority. Atopic March refers to the sequential clinical progression from atopic dermatitis (eczema) to food allergy, allergic rhinitis, and asthma. Westernized Shift is a dietary transition toward energy-dense, highly processed foods, which has been identified as a primary driver of the region’s allergy epidemic. Significance denotes statistical significance where *p* ≤ 0.05. Self-report bias indicates data derived from participant surveys rather than objective clinical measures such as skin prick tests (SPT) or oral food challenges (OFC).

### 3.5 Milk allergy in Saudi population

Research into milk allergy across Saudi Arabia confirms it is a major and growing health issue, particularly for children. Findings from various cities show that allergy to cow’s milk protein is widespread, yet families and the healthcare system face challenges in diagnosis, daily management, and emergency readiness.

Detailed local research, such as a 2025 study in Abha, compared young children with confirmed milk allergy to healthy peers. It found that the highest risk was having a sibling with the same condition. Infant feeding choices mattered: using only cow’s milk formula raised the risk, while combining it with other feeding methods had a protective effect. Parents were generally aware of what causes allergies, but knew less about practical steps, like which alternative formulas to use. Though well-designed, studies like this one focused on a single area show why broader research is needed (26).

A finding from a 2025 national survey highlights a serious gap in safety. It revealed that only about one in four parents of a child with food allergies had an epinephrine auto-injector, the essential treatment for a severe reaction. Families were more likely to have this device if the child had previously suffered breathing or stomach problems during a reaction, or if they regularly saw an allergy doctor. The fact that most families did not have one, despite the known risks, points to a system-wide problem in education and access (16).

Earlier summaries of global and local knowledge, like a 2021 review, established that milk allergy affects an estimated 2–3% of infants. Reactions can be rapid (IgE-mediated) or delayed (non-IgE-mediated), with family history and early exposure to cow’s milk being key risks. This review specifically noted a shortage of Saudi-based studies on the delayed, IgG-related type of milk sensitivity (27). However, IgG food testing is clinically discouraged because it indicates past exposure rather than a true allergic reaction.

Acting on this identified need for local data, a 2023 study from Jeddah examined both types of reactions in patients. It reported that immediate (IgE) allergies were often triggered by different proteins in adults versus children. More notably, it found that delayed (IgG) sensitivity to milk appeared in most adults and every child in their sample. The study also noted that having a pet might slightly lower the risk, an observation that aligns with the idea that early exposure to certain germs can train the immune system (28).

### 3.6 Dietary management and nutritional adequacy in food-allergic patients

The cornerstone of food allergy management remains dietary elimination of trigger foods. While this approach prevents acute reactions, excluding multiple staple foods carries nutritional risks, particularly for children during their growth periods. A 2023 Riyadh study demonstrated this directly: children with confirmed food allergies who eliminated eggs, milk, and wheat simultaneously showed significantly lower height-for-age and body mass index z-scores than non-allergic peers, with the effect most pronounced in boys (*p* ≤ 0.05) (4). So these results indicate that diet restriction without a proper nutritional plan is not beneficial; on the contrary, it may lead to stunted growth due to a deficiency of key micronutrients.

Cow’s milk protein allergy (CMPA) poses the most clinically complex nutritional challenge in the Saudi pediatric population, given that cow’s milk is both the most prevalent allergen and a primary source of calcium, vitamin D, protein, and energy in early childhood. Standard management requires substitution with an appropriate hypoallergenic formula: extensively hydrolysed casein formula (eHCF) or, for infants with multiple food protein intolerance, an amino acid-based formula (AAF). A local 2025 case–control study in Abha found that relying exclusively on cow’s milk formula increased CMPA risk, whereas combining feeding strategies offered a protective effect, suggesting that nutritional diversification from infancy has both allergenic and adequacy benefits (26). An important local alternative under investigation is camel milk: its distinct protein profile, specifically the absence of beta-lactoglobulin, results in low cross-reactivity with cow’s milk proteins, and preliminary Saudi data from a study showed that a majority of CMPA infants tolerate it without reaction (6). However, camel milk does not meet the full nutritional specification of an infant formula and should not replace prescribed hypoallergenic formulas without dietetic supervision.

Vitamin D deficiency represents a compounding nutritional concern uniquely relevant to Saudi food-allergic patients. Saudi Arabia paradoxically reports high rates of vitamin D deficiency despite abundant sunlight, attributable to cultural dress practices, indoor lifestyle, and low dietary intake. Vitamin D insufficiency is independently associated with increased risk of sensitisation and atopic disease, and its deficiency in food-allergic children who are simultaneously avoiding fortified dairy products creates a dual pathway for inadequacy (4). Current evidence supports routine vitamin D monitoring in food-allergic Saudi children and supplementation as part of the elimination diet protocol. Similarly, egg elimination, which affects up to 22% of adults in some local surveys, removes a key source of choline, riboflavin, and high-biological-value protein from the diet; sesame elimination, relevant to the Saudi population given its dietary ubiquity in tahini and halwa, removes a significant source of calcium, iron, and monounsaturated fatty acids. These micronutrient gaps are rarely addressed in the Saudi clinical literature, exposing an area for dietetic research and protocol development.

There is also emerging evidence that dietary patterns, independent of specific allergen avoidance, modulate immune function and allergy risk in the Saudi population. The ongoing shift toward a “Westernized” dietary pattern, characterized by high intake of ultra-processed foods, refined carbohydrates, and saturated fats alongside reduced fiber, fruits, and vegetables, is associated with gut microbiome dysbiosis and heightened Th2-type immune responses that favor allergic sensitization (17). Conversely, dietary patterns rich in omega-3 fatty acids, prebiotic fibers, and fermented foods are hypothesized to support regulatory immune pathways and reduce atopic burden. In the Saudi context, this suggests a dual nutritional strategy is warranted: (1) allergen-specific elimination diets that are nutritionally complete and dietitian-supervised, with periodic re-evaluation for tolerance development, particularly in children under five; and (2) population-level dietary guidance promoting anti-inflammatory eating patterns that may reduce the background risk of new sensitisations. Integration of registered dietitians into allergy care teams, currently inconsistent across Saudi healthcare centers, is essential to operationalize both strategies safely.

Although cow’s milk, eggs, seafood, and wheat were the allergens most frequently investigated in Saudi studies, limited data were available regarding peanut and tree nut allergy. This represents an important knowledge gap because peanuts and tree nuts are among the leading causes of severe and persistent food allergy worldwide. Future Saudi epidemiological studies should specifically evaluate these allergens.

### 3.7 Food allergy management and Saudi food and drug authority (SFDA) regulatory initiatives

While clinical interventions address individual cases of food allergy, population-level prevention requires robust regulatory policy. As of 2025, the SFDA has established a multi-faceted regulatory framework aimed at reducing the incidence of accidental allergen exposure through standardized labeling, food service regulation, and public health education (29).

#### 3.7.1 Mandatory allergen declaration on packaged foods

The SFDA requires mandatory declaration of major food allergens on prepackaged food labels, aligned with Codex Alimentarius guidelines and regulations in jurisdictions such as the United States and European Union (1). The specified allergens include cereals containing gluten, crustaceans, eggs, fish, peanuts, milk, tree nuts, soybeans, sesame seeds, and sulfites (≥10 mg/kg). Of particular epidemiological relevance is the inclusion of sesame, a high-prevalence allergen in Middle Eastern populations due to its dietary ubiquity in products such as tahini and halwa. This reflects the adaptation of international standards to regional consumption patterns and public health needs. Despite their inclusion in SFDA allergen labeling regulations, epidemiological data on peanut and tree nut allergy in Saudi Arabia remain limited.

#### 3.7.2 Regulation of precautionary allergen labeling (PAL)

The Precautionary Allergen Labeling (e.g., “may contain,” “processed in a facility with”)requires declaration when an allergen is incorporated as an ingredient. However, labeling for cross-contact due to shared production lines remains less standardized. Nevertheless, overuse of such warnings can lead to unnecessary dietary restriction, reduced quality of life, and diminished label credibility (30). Further, the absence of evidence-based thresholds for actionable cross-contact contributes to consumer confusion and risk misinterpretation.

#### 3.7.3 Food service sector regulation

The SFDA has initiated steps to regulate allergen management within the food service industry. The measures include mandatory allergen awareness training for food handlers, requirements for major allergen disclosure on menus, and implementation of cross-contamination prevention protocols.

#### 3.7.4 Public awareness and education initiatives

The SFDA, in collaboration with the Ministry of Health, has engaged in public health campaigns to improve allergen knowledge among consumers, educators, and food industry professionals. Evidence suggests the potential efficacy of such initiatives. A 2022 survey in Saudi Arabia indicated that information from official government sources and healthcare providers was positively correlated with higher knowledge scores among caregivers of children with food allergy (20). Targeted campaigns focusing on label literacy, anaphylaxis recognition, and emergency management could address the persistent awareness gaps identified in national surveys (25).

## 4 Discussion

The literature related to food allergy in Saudi Arabia is growing, but it does not provide a full understanding of the problem. One of the major limitations is that most of the available data have been obtained through surveys using self-reported questionnaires. This methodological limitation has significant implications for the reliability of prevalence estimates across the literature. Of the 20 studies included in this review, 9 (45%) relied exclusively on self-reported questionnaires or parental proxy reports without any objective diagnostic confirmation. Only 6 studies used objective immunological testing. However, while serum-specific IgE testing is widely accepted as part of food allergy diagnosis, food-specific IgG testing is not recommended for diagnostic purposes and should be interpreted cautiously as a marker of exposure rather than disease [Alkhateeb, 2020 (10); Shakoor et al., 2016 (11); Atwah and Koshak, 2024 (19); Baghlaf et al., 2021, 2023 (27, 28); Aburiziza, 2026 (6)]. Only 2 studies (6.5%) employed skin prick testing (SPT) as a confirmatory diagnostic tool [Alqurashi et al., 2025 (26); Aburiziza, 2026 (6)]. Notably, none of the included Saudi studies employed oral food challenge (OFC) or double-blind placebo-controlled food challenge (DBPCFC), which remain the accepted gold-standard methods for confirming food allergy. This represents one of the most important limitations of the current evidence base. Another important limitation is the lack of detailed clinical history in many of the included studies. Information regarding symptom reproducibility, timing of reactions, quantity of allergen exposure, and response to elimination or reintroduction was frequently unavailable. Because clinical history is central to food allergy diagnosis, the absence of these data limits the interpretation of laboratory findings. Furthermore, positive serum-specific IgE results indicate sensitization rather than clinically confirmed allergy. In the absence of supportive clinical history and challenge testing, sensitization data should not be interpreted as definitive evidence of food allergy. This observation is consistent with findings from the EuroPrevall project and other international studies, which demonstrated that sensitization rates frequently exceed challenge-confirmed food allergy prevalence. Similar patterns appear evident within the Saudi literature, where laboratory sensitization and self-reported allergy are substantially more common than clinically confirmed food allergy.

Diagnostic approaches varied substantially among studies and included self-reported questionnaires, physician diagnosis, serum-specific IgE testing, skin prick testing, and food-specific IgG assays. This diagnostic heterogeneity limits direct comparison between studies and may contribute to variability in reported prevalence estimates. This heavy reliance on self-reported data means that figures cited in this review, such as the 19–20% adult prevalence, likely represent perceived food hypersensitivity rather than clinically confirmed IgE-mediated food allergy. However, this reported prevalence likely reflects perceived food hypersensitivity rather than clinically confirmed, IgE-mediated food allergy. Because the majority of the underlying data relies on self-reported surveys, this figure should be interpreted as an upper bound that highly likely overestimates the true clinical prevalence of food allergies in this population. Another limitation is the absence of long-term birth cohort studies within the Kingdom. Most existing data provide only short-term cross-sectional data. In addition, several included studies employed retrospective designs, which are inherently susceptible to selection bias, incomplete data collection, and inability to establish temporal relationships between exposure and outcome. Consequently, causal inferences regarding food allergy risk factors should be interpreted cautiously. A further limitation of the available literature is the inconsistent reporting of allergen-specific diagnostic thresholds. Most studies reported the presence of sensitization or positive immunological findings without providing standardized cutoff values for common allergens such as milk, egg, fish, peanut, or tree nuts. This limited comparison across studies and prevented assessment of clinically meaningful sensitization levels. Because this review was based on published evidence rather than individual patient records, direct correlation between laboratory findings and detailed clinical histories was not possible. Future studies in Saudi Arabia should integrate clinical history, symptom reproducibility, laboratory investigations, and oral food challenge confirmation to improve diagnostic accuracy and distinguish sensitization from clinically relevant food allergy.

Tracking children from birth is essential to understand the “atopic march” the natural progression where conditions like eczema and food allergy in infancy often lead to asthma and allergic rhinitis later in childhood. Future studies must investigate how this genetic background influences the development, severity, and progression of allergic diseases compared to other populations. An important limitation of several studies included in this review is their reliance on food-specific IgG assays. According to current EAACI and NIAID recommendations, food-specific IgG antibodies are not diagnostic of food allergy and may represent normal immune responses associated with exposure and tolerance. Therefore, findings based on IgG reactivity should not be interpreted as evidence of allergic disease without corroborating clinical history and validated diagnostic testing. An additional methodological limitation is the absence of prospective protocol registration. Although the review followed PRISMA 2020 guidance, registration in databases such as PROSPERO would have further enhanced transparency and reduced the potential for reporting bias. Furthermore, a formal GRADE assessment was not performed because of the substantial heterogeneity of study designs and outcomes, which precluded quantitative synthesis. Nevertheless, a structured risk-of-bias assessment was conducted to support the interpretation of the evidence. Additional methodological limitations should be considered. Restricting inclusion to English-language publications may have resulted in language bias, although most biomedical research indexed in the selected databases is published in English. Furthermore, while grey literature sources and regulatory documents were screened, unpublished studies and local reports may not have been fully captured. Consequently, some degree of publication bias cannot be excluded.

A notable characteristic of the available literature is its substantial heterogeneity. The evidence base includes clinical investigations, prevalence surveys, awareness assessments, laboratory-based studies, review articles, and regulatory documents, each addressing different aspects of food allergy. Consequently, direct comparison between studies is often difficult, and conclusions should be interpreted within the context of the specific evidence type from which they were derived. The narrative synthesis approach adopted in this review was therefore considered the most appropriate method for integrating these diverse sources of evidence. Interpretation of food allergy outcomes also varied considerably between studies. Some investigations reported self-perceived food allergy, whereas others reported sensitization markers or clinically diagnosed disease. Therefore, findings should be interpreted within the context of the diagnostic methodology used in each study. Most available evidence originates from urban centers, including Riyadh, Jeddah, Makkah, and Abha. Consequently, rural populations remain underrepresented. Differences in environmental exposures, dietary patterns, healthcare accessibility, and lifestyle factors may contribute to rural–urban variation in allergy prevalence, but current evidence is insufficient to evaluate these relationships. A unified system through a standardized national surveillance program or registry would provide reliable, ongoing data on prevalence, triggers, and outcomes across all regions. Finally, some studies revealed that high awareness of allergy severity does not translate into preparedness for emergencies. Future work must move beyond simply measuring knowledge. It should identify the specific barriers that prevent families from obtaining and using this lifesaving medication.

## 5 Conclusion

The available evidence suggests that food sensitization and self-reported food allergy are common in Saudi Arabia; however, the true prevalence of clinically confirmed food allergy remains uncertain because most studies relied on questionnaires, sensitization markers, or retrospective clinical assessment rather than oral food challenge confirmation. The genetic factors, regional dietary habits, and a modernizing environment contribute to food allergy, which is similar to the global trend but with local distinctions. Though the SFDA recommendation of food labeling as a preventive measure is crucial, but better food allergy cannot be prevented from labeling alone; an equal focus on the healthcare system is required. The evidence gathered in this review shows an urgent need for standardized national guidelines for diagnosis and management to ensure consistent care. Along with this, efforts to improve education for healthcare providers, parents, and educators on emergency preparedness should be made. The dietary elimination requires a dietitian-monitored elimination protocol and routine dietary monitoring in Saudi Arabia. The findings should be interpreted in light of the predominantly moderate quality of the available evidence, which is largely based on cross-sectional and self-reported studies. Finally, foundational research through longitudinal studies, a national registry, and investigations into genetic and immunological mechanisms is essential to fill gaps.

## Data Availability

The original contributions presented in the study are included in the article/supplementary material, further inquiries can be directed to the corresponding author.
